# Maternal gestational weight gain and offspring vitamin D level: a multicentre retrospective cohort study

**DOI:** 10.3389/fendo.2026.1936050

**Published:** 2026-10-08

**Authors:** Yan Zhao, Qian Li, Hong Zhu, Wen Zheng, Yufei Ni, Aiping Wu, Kan Ye, Xinye Jiang, Guoqin Liu, Yelin Bao, Guoqiang Yang, Li Zhang, Hongxia Qi, Heyun Lv, Rui Qin, Hong Cao

**Affiliations:** 1 Department of Nutrition, Affiliated Hospital of Jiangnan University, Wuxi, China; 2 Wuxi School of Medicine, Jiangnan University, Wuxi, China; 3 Department of Clinical Nutrition, Jiangsu Province Hospital, the First Affiliated Hospital with Nanjing Medical University, Nanjing, Jiangsu, China; 4 Institute of Future Food Technology, JITRI, Yixing, China; 5 Department of Clinical Nutrition, The Affiliated Suqian Hospital of Xuzhou Medical University, Nanjing Drum Tower Hospital Group Suqian Hospital, Suqian, China; 6 Department of Child Health Care, Yancheng Maternity and Child Health Care Institute, Yancheng, China; 7 Department of Child Health Care, Nantong Maternity and Child Health Care Hospital, Nantong, China; 8 Department of Child Health Care, Xinghua Maternity and Child Health Care Hospital, Xinghua, China; 9 Department of Child Health Care, The Affiliated Suzhou Hospital of Nanjing Medical University, Suzhou Municipal Hospital, Gusu School, Nanjing Medical University, Suzhou, China; 10 Department of Child Health Care, Wuxi Maternity and Child Health Care Hospital, Wuxi, China; 11 Department of Child Health Care, Dafeng Maternity and Child Health Care Hospital, Dafeng, China; 12 Department of Child Health Care, Drum Tower Maternity and Child Health Care Institute, Nanjing, China; 13 Department of Child Health Care, Kunshan Maternity and Child Health Care Institute, Kunshan, China; 14 Department of Child Health Care, Huai’an Maternity and Child Health Care Hospital, Huai’an, China; 15 Department of Child Health Care, Xuzhou Children’s Hospital, Xuzhou, China; 16 Department of Child Health Care, Jiangning Maternity and Child Health Care Institute, Nanjing, China; 17 Department of Child Health Care, Jiangsu Women and Children Health Hospital, Women and Child Branch Hospital of Jiangsu Province Hospital, The First Affiliated Hospital with Nanjing Medical University, Nanjing, China

**Keywords:** 25-hydroxyvitamin D, gestational weight gain, offspring, retrospective cohort study, vitamin D deficiency

## Abstract

**Background:**

This study examined the association between maternal gestational weight gain (GWG) and offspring vitamin D levels.

**Methods:**

In this retrospective cohort study, 0–5-year-old children attending routine health checks at 12 centres across 10 cities in Jiangsu Province (April 2014–March 2015) were enrolled via stratified random cluster sampling. Serum 25(OH)D was measured by ELISA.

**Results:**

Among 1,984 mother–offspring pairs, GWG ≤10 kg was associated with an increased risk of vitamin D deficiency (<50 nmol/L) in the unadjusted model (*OR* = 1.550, 95% *CI*: 1.196–2.008), and this association remained significant in Model 1 (*OR* = 1.820, 95% *CI*: 1.357–2.441), Model 2 (*OR* = 1.545, 95% *CI*: 1.060–2.253), and Model 3 (*OR* = 1.882, 95% *CI*: 1.228–2.883). For vitamin D insufficiency (50–<75 nmol/L), GWG >19 kg was associated with an increased risk in the unadjusted model (*OR* = 1.309, 95% *CI*: 1.026–1.670) and in Model 1 (*OR* = 1.312, 95% *CI*: 1.021–1.685), Model 2 (*OR* = 1.872, 95% *CI*: 1.305–2.684), and Model 3 (*OR* = 1.609, 95% *CI*: 1.054–2.456). This association was statistically significant in Model 3 but was no longer significant after FDR correction.

**Conclusion:**

To our knowledge, this is the first multicentre study to report an association between GWG ≤10 kg and an increased risk of vitamin D deficiency in offspring, and between GWG >19 kg and an increased risk of vitamin D insufficiency. However, the latter association did not remain significant after FDR correction in sensitivity analyses based on Model 3 and requires confirmation in further studies.

## 1 Introduction

Pregnancy is a critical window for foetal development, where maternal nutrition and metabolism can profoundly influence offspring growth and long-term health. Gestational weight gain (GWG), reflecting increases in uterine size, placenta, amniotic fluid, blood volume, breast tissue, fat stores, and foetal growth, is consistently associated with adverse perinatal outcomes when insufficient or excessive (1–4). Beyond shaping foetal intrauterine growth, GWG may also exert lasting effects on postnatal development by altering maternal metabolic status and nutrient transport efficiency (5, 6).

Among the various adverse outcomes, vitamin D deficiency in offspring is a major public health concern. In addition to its established role in calcium and phosphorus homeostasis, vitamin D is essential for immune function, cell differentiation and endocrine regulation. Infancy and early childhood are marked by a high prevalence of vitamin D deficiency, which is associated with immediate consequences such as rickets and impaired immunity, as well as an elevated long-term risk of chronic disease (7). In Jiangsu Province, China, 30.1% of children aged 0–5 years have vitamin D insufficiency or deficiency (8), underscoring the urgency of this issue.

Although maternal characteristics are increasingly recognised as key determinants of offspring vitamin D levels, most studies have focused on pre-pregnancy body mass index (BMI), with relatively little attention paid to GWG, which is more amenable to clinical intervention (9, 10). To date, only one small study (n = 59) has preliminarily examined the association between GWG and offspring vitamin D status, and its limited sample size restricts both statistical power and generalisability (11). Given the high global prevalence of vitamin D deficiency in mothers and infants and its substantial public health implications, clarifying the relationship between GWG and offspring vitamin D levels is essential for optimising perinatal care. To address this gap, the present study uses large-scale, multicentre retrospective cohort data from China to systematically investigate the association between maternal GWG and infant vitamin D levels, with a particular focus on identifying potential risk groups to inform gestational weight management and early-life nutritional interventions.

## 2 Methods

### 2.1 Participants and study design

This was a large-scale multicentre retrospective cohort study. From April 2014 to March 2015, Jiangsu Province was divided into four regions (Nanjing, Southern Jiangsu, Central Jiangsu, and Northern Jiangsu) using stratified cluster sampling, with 3–4 centres randomly selected from each region. Children aged 0–5 years, corresponding to 0–71 months, who underwent routine health checkups were recruited from 10 cities across Jiangsu Province (**Figure 1**). At the child health visit, maternal exposure information across different periods, including pre-pregnancy, pregnancy, pre-delivery, and lactation, was retrospectively collected. The present analysis focused on pregnancy-related exposures, with gestational weight gain as the primary exposure of interest. Pre-pregnancy weight and pre-delivery weight were extracted from routine antenatal care records, and gestational weight gain was calculated as the difference between these two objectively documented measurements. The effect of different categories of gestational weight gain (calculated as the difference between pre-delivery and pre-pregnancy weight) on offspring vitamin D levels was analysed.

**Figure 1:**
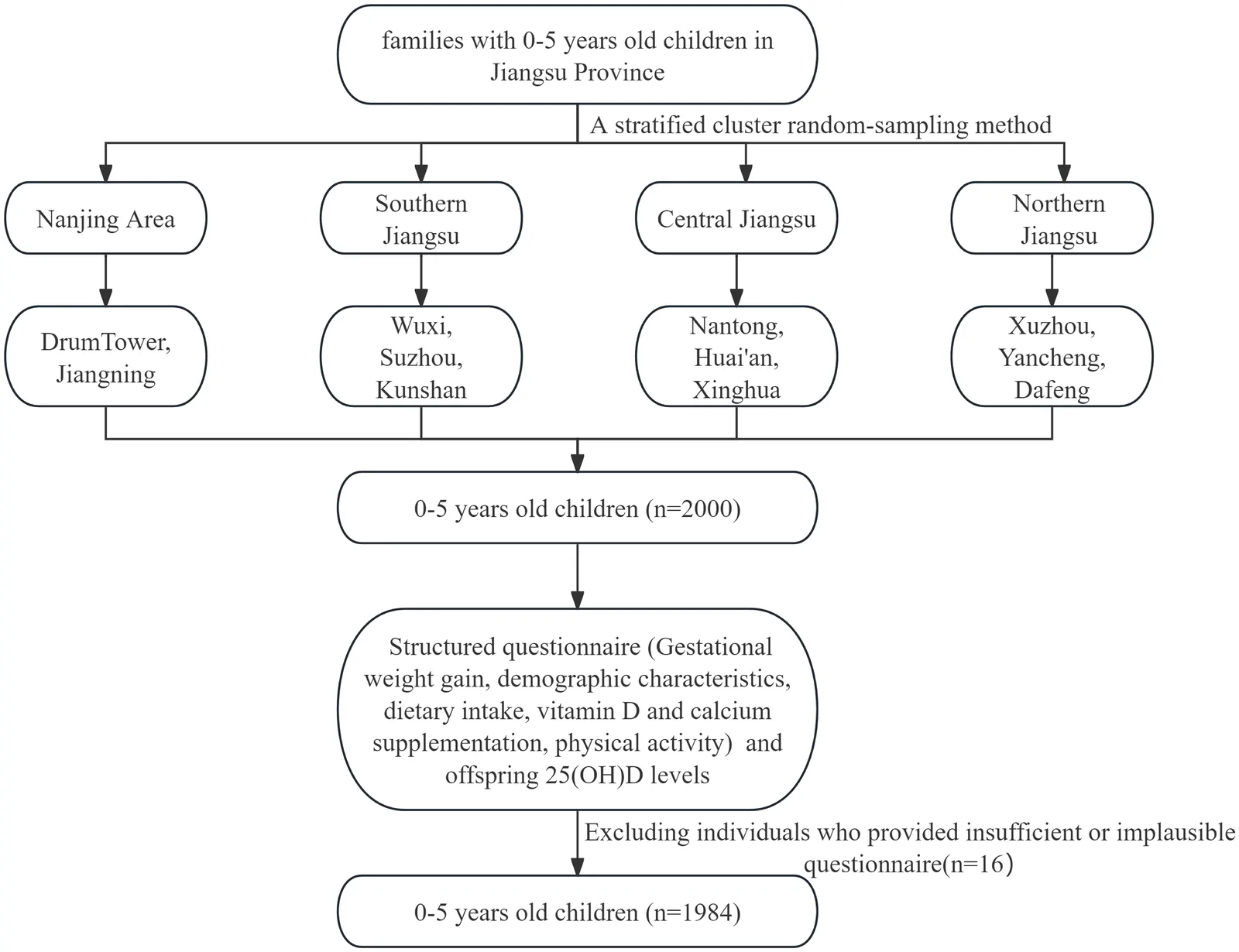
Participant flowchart of the study.

### 2.2 Questionnaire survey

We collected data on children and mothers via face-to-face interviews using a structured questionnaire, as detailed in our prior publication. This instrument captured key demographic and perinatal factors: child’s age, sex, maternal gravidity/parity, gestational age, delivery mode, birth weight, birth season, and household registration (urban/rural). Maternal lifestyle factors during pregnancy were also systematically recorded (12), covering dietary intake, physical activity, and the use of vitamin D and calcium supplements. Child lifestyle factors were collected, including dietary intake, physical activity, and the use of vitamin D and calcium supplements. These variables have been described and analysed in detail in our previous publication based on the same cohort (8).

### 2.3 Gestational weight gain and vitamin D classification

#### 2.3.1 Gestational weight gain

In accordance with the Chinese health industry standard WS/T 801-2022 (13), pre-pregnancy weight is defined as the average body weight within the three months before pregnancy; pre-delivery weight is defined as the last measured body weight within one week before delivery; gestational weight gain is defined as the value obtained by subtracting pre-pregnancy weight from pre-delivery weight. In this study, body weight at the two time points of pre-pregnancy and pre-delivery were obtained separately to calculate gestational weight gain, representing the cumulative weight change over the entire pregnancy period.

#### 2.3.2 Vitamin D classification

Vitamin D level was classified according to serum 25-hydroxyvitamin D [25(OH)D] concentration as follows (14): deficiency, <50 nmol/L (20 ng/mL); insufficiency, 50–<75 nmol/L (20–< 30 ng/mL); and sufficiency, ≥ 75 nmol/L (≥ 30 ng/mL). In addition, the composite outcome of vitamin D deficiency or insufficiency was defined as 25(OH)D <75 nmol/L, combining the deficiency and insufficiency categories.

### 2.4 Vitamin D measurement

Blood samples (120 μL) were collected via finger prick and stored at 4 °C. Serum 25(OH)D concentrations were analysed within one week using an enzyme-linked immunosorbent assay kit supplied by IDS Ltd. (Tyne & Wear, United Kingdom). Technicians performed all assays following standardised manufacturer protocols, with full documentation maintained for quality control.

### 2.5 Statistical analysis

Continuous variables were compared using Mann-Whitney U or Kruskal-Wallis tests, and categorical variables using χ² tests. All analyses were performed using SPSS (version 25.0) and R (version 4.2.1), with two-tailed *P* < 0.05 considered statistically significant.

In this study, the distribution of GWG showed that values remained at 10 kg from the 10th percentile through the 19.5th percentile, indicating a marked concentration at this value in the lower tail of the distribution; the 50th percentile (median) was 15 kg, and the 75th percentile was 19 kg.

To explore the shape of the association between continuous GWG and offspring vitamin D levels, we fitted multiple curve models with serum 25(OH)D as a continuous outcome; the best-fitting model was selected via visual inspection and adjusted R². The exploratory curve fitting analysis suggested a change in the association pattern at approximately 10 kg of GWG. Accordingly, based on the above distributional characteristics combined with the curve fitting results, GWG was categorised into three groups (≤10 kg, 10 < GWG ≤ 19 kg, and >19 kg) for subsequent logistic regression analyses. These cut-offs are considered exploratory rather than definitive biological thresholds.

Child age was categorised into six groups (0–11, 12–23, 24–35, 36–47, 48–59, and 60–71 months) to flexibly capture age-related variations in vitamin D levels and lifestyle factors across early childhood without imposing a linear functional form, as vitamin D metabolism and lifestyle factors vary substantially across these developmental stages.

Two separate binary logistic regression analyses were performed to examine the associations between GWG categories and offspring vitamin D outcomes: (1) deficiency (<50 nmol/L) versus non-deficiency (≥50 nmol/L); and (2) deficiency or insufficiency (<75 nmol/L) versus sufficiency (≥75 nmol/L). For each analysis, GWG was categorised into three groups (≤10 kg, 10 < GWG ≤ 19 kg, and >19 kg), with 10 < GWG ≤ 19 kg as the reference. In addition, multinomial logistic regression was performed with offspring vitamin D status as a three-category outcome (deficiency, <50 nmol/L; insufficiency, 50–<75 nmol/L; sufficiency, ≥75 nmol/L as the reference category) to evaluate the associations between GWG categories and each vitamin D outcome separately.

Three adjustment models were constructed. Model 1 adjusted for demographic characteristics (household registration, child age group, number of pregnancies, investigation season, and regional distribution); Model 2 adjusted for maternal lifestyle factors during pregnancy (timing and use of vitamin D and calcium supplements during pregnancy, daily milk intake, fish and shrimp intake, and maternal outdoor activity time during pregnancy); Model 3 adjusted for child lifestyle factors (daily milk intake (binary classification), timing of the first vitamin D supplementation after birth, age at first complementary feeding (months), vitamin D and calcium supplementation within the past 3 months, frequency of fish and shrimp intake, and child daily outdoor activity time).

To further explore the shape of the association between GWG and offspring vitamin D deficiency without imposing a predefined functional form, we performed restricted cubic spline (RCS) regression as a sensitivity analysis, with vitamin D deficiency (<50 nmol/L) as a binary outcome and GWG as a continuous predictor, using 4 knots at the 5th, 35th, 65th, and 95th percentiles, adjusted for the same covariates as in Model 1. Results are presented in **Supplementary Figure S1**. Model 3 adjusted for child lifestyle factors, and the results are presented in **Tables 1A**, **B**. Additional sensitivity analyses based on Model 3 are presented in **Supplementary Tables S1a, b**. For these sensitivity analyses, FDR correction was applied using the Benjamini–Hochberg method.

**Table 1a:** Odds ratios (95% CIs) for the association between maternal GWG categories and offspring vitamin D outcomes: binary logistic regression analyses.

Gestational weight gain OR (95% *CI*)			
Variable	GWG ≤10 kg	10 < GWG ≤ 19 kg	GWG >19 kg
Offspring’s 25(OH)D <50 nmol/L			
Unadjusted	**1.550 (1.196, 2.008)	Ref.	0.996 (0.771, 1.287)
Model 1	**1.820 (1.357, 2.441)	Ref.	1.233 (0.927, 1.640)
Model 2	*1.545 (1.060, 2.253)	Ref.	0.759 (0.509, 1.132)
Model 3	**1.882 (1.228, 2.883)	Ref.	1.119 (0.706, 1.775)
Offspring’s 25(OH)D < 75 nmol/L			
Unadjusted	1.208 (0.957, 1.525)	Ref.	1.227 (0.990, 1.520)
Model 1	1.186 (0.923, 1.523)	Ref.	*1.344 (1.067, 1.693)
Model 2	*1.451 (1.027, 2.050)	Ref.	*1.472 (1.067, 2.031)
Model 3	1.278 (0.880, 1.856)	Ref.	1.349 (0.939, 1.937)

**P < 0.01; *P < 0.05.

Two separate binary logistic regression analyses were performed. For each analysis, the reference category for gestational weight gain (GWG) was 10 < GWG ≤ 19 kg. Analysis 1: outcome = 25(OH)D <50 nmol/L (deficiency), reference = ≥50 nmol/L; Analysis 2: outcome = 25(OH)D <75 nmol/L (deficiency or insufficiency), reference = ≥75 nmol/L. Model 1 adjusted for demographic characteristics (household registration, child age group, number of pregnancies, investigation season, and regional distribution); Model 2 adjusted for maternal lifestyle factors during pregnancy, including timing and use of vitamin D and calcium supplements during pregnancy, daily milk intake, fish and shrimp intake, and maternal outdoor activity time during pregnancy; Model 3 adjusted for child lifestyle factors, including daily milk intake (binary classification), timing of the first vitamin D supplementation after birth, age at first complementary feeding (months), vitamin D and calcium supplementation within the past 3 months, frequency of fish and shrimp intake, and child daily outdoor activity time.

**Table 1b:** Odds ratios (95% CIs) for the association between maternal GWG categories and offspring vitamin D outcomes: multinomial logistic regression analyses.

Gestational weight gain OR (95% *CI*)			
Variable	GWG ≤10 kg	10 < GWG ≤ 19 kg	GWG >19 kg
Offspring’s 25(OH)D <50 nmol/L			
Unadjusted	**1.532 (1.157, 2.027)	Ref.	1.115 (0.845, 1.471)
Model 1	**1.537 (1.143, 2.066)	Ref.	1.048 (0.786, 1.396)
Model 2	*1.666 (1.107, 2.508)	Ref.	0.994 (0.649, 1.525)
Model 3	*1.948 (1.161, 3.268)	Ref.	1.346 (0.779, 2.325)
50 ≤ Offspring’s 25(OH)D <75 nmol/L			
Unadjusted	0.970 (0.732, 1.286)	Ref.	*1.309 (1.026, 1.670)
Model 1	0.939 (0.703, 1.254)	Ref.	*1.312 (1.021, 1.685)
Model 2	1.228 (0.809, 1.863)	Ref.	**1.872 (1.305, 2.684)
Model 3	1.127 (0.707, 1.796)	Ref.	*1.609 (1.054, 2.456)

**P < 0.01; *P < 0.05.

Multinomial logistic regression was performed with offspring vitamin D status as a three-category outcome (deficiency, <50 nmol/L; insufficiency, 50–<75 nmol/L; sufficiency, ≥75 nmol/L as the reference category). The reference category for gestational weight gain (GWG) was 10 < GWG ≤ 19 kg. Model 1 adjusted for demographic characteristics (household registration, child age group, number of pregnancies, investigation season, and regional distribution); Model 2 adjusted for maternal lifestyle factors during pregnancy, including timing and use of vitamin D and calcium supplements during pregnancy, daily milk intake, fish and shrimp intake, and maternal outdoor activity time during pregnancy; Model 3 adjusted for child lifestyle factors, including daily milk intake (binary classification), timing of the first vitamin D supplementation after birth, age at first complementary feeding (months), vitamin D and calcium supplementation within the past 3 months, frequency of fish and shrimp intake, and child daily outdoor activity time.

## 3 Results

### 3.1 Analysis of GWG in pregnant women with different characteristics

This study included 1,984 mother–offspring pairs categorised into three groups based on GWG: ≤10 kg (19.5%), 10 < GWG ≤ 19 kg (55.8%), and >19 kg (24.7%). As shown in **Table 2**, significantly higher proportions of GWG >19 kg were observed amongst mothers with the following characteristics: survey in winter, child age 60–71 months, first pregnancy, first parity, preterm birth, caesarean section, birth weight ≥4000 g, residence in Nanjing, calf cramps during pregnancy, use of vitamin D and calcium supplements during pregnancy, initiation of supplementation within 5 gestational weeks, daily milk intake ≥200 mL, and fish/shrimp intake <4 times per week (*P* < 0.05).

**Table 2:** Maternal demographic and perinatal characteristics stratified by gestational weight gain categories and separately by offspring vitamin D status.

Classification	N	Gestational weight gain						N	Offspring 25(OH)D, nmol/L		Offspring vitamin D levels and deficiency			
		GWG ≤10 kg	10 < GWG ≤ 19 kg	GWG >19 kg	*p*	*P50 (P25–P75)*	*p*		*P*50(25- *P*75)	*p*	VitD<50 nmol/L	50≤VitD<75 nmol/L	VitD≥75 nmol/L	*p*
		N (%)	N (%)	N (%)							N (%)	N (%)	N (%)	
Household registration														
Town	1355	264 (19.5%)	765 (56.5%)	326 (24.1%)	0.531	15.0 (12.0–19.0)	0.540	1355	69.9 (51.6–93.5)	0.781	304 (22.4%)	441 (32.5%)	610 (45.0%)	0.016
Countryside	628	123 (19.6%)	340 (54.1%)	165 (26.3%)		15.0 (12.0–20.0)		628	72.2 (49.1–96.5)		166 (26.4%)	167 (26.6%)	295 (47.0%)	
Sex														
Boy	1036	195 (18.8%)	573 (55.3%)	268 (25.9%)	0.429	15.0 (12.0–20.0)	0.594	1036	71.2 (53.1–93.5)	0.632	227 (21.9%)	337 (32.5%)	472 (45.6%)	0.067
Girl	948	192 (20.3%)	533 (56.2%)	223 (23.5%)		15.0 (12.0–18.0)		948	70.4 (49.4–96.0)		243 (25.6%)	271 (28.6%)	434 (45.8%)	
Season of survey														
Spring	387	80 (20.7%)	206 (53.2%)	101 (26.1%)	0.003	15.0 (12.0–20.0)	0.379	387	65.0 (44.4–88.9)	<0.001	125 (32.3%)	120 (31.0%)	142 (36.7%)	<0.001
Summer	1082	194 (17.9%)	615 (56.8%)	273 (25.2%)		15.0 (12.5–20.0)		1082	73.9 (49.0–99.0)		282 (26.1%)	272 (25.1%)	528 (48.8%)	
Autumn	204	35 (17.2%)	134 (65.7%)	35 (17.2%)		15.0 (12.5–17.0)		204	81.0 (64.7–99.2)		21 (10.3%)	61 (29.9%)	122 (59.8%)	
Winter	311	78 (25.1%)	151 (48.6%)	82 (26.4%)		15.0 (10.0–20.0)		311	68.2 (55.9–80.8)		42 (13.5%)	155 (49.8%)	114 (36.7%)	
Child age														
0–11 months	912	159 (17.4%)	527 (57.8%)	226 (24.8%)	0.009	15.0 (12.1–19.0)	0.039	912	76.0 (53.1–99.0)	<0.001	202 (22.1%)	239 (26.2%)	471 (51.6%)	<0.001
12–23 months	839	168 (20.0%)	473 (56.4%)	198 (23.6%)		15.0 (12.0–18.0)		839	71.0 (52.6–94.3)		178 (21.2%)	279 (33.3%)	382 (45.5%)	
24–35 months	52	14 (26.9%)	26 (50.0%)	12 (23.1%)		15.0 (10.0–18.3)		52	60.1 (45.3–82.7)		14 (26.9%)	21 (40.4%)	17 (32.7%)	
36–47 months	27	7 (25.9%)	14 (51.9%)	6 (22.2%)		15.0 (10.0–17.5)		27	54.6 (44.4–77.6)		10 (37.0%)	10 (37.0%)	7 (25.9%)	
48–59 months	76	22 (28.9%)	37 (48.7%)	17 (22.4%)		14.5 (10.0–17.9)		76	48.8 (39.6–63.6)		41 (53.9%)	26 (34.2%)	9 (11.8%)	
60–71 months	78	17 (21.8%)	29 (37.2%)	32 (41.0%)		15.8 (12.0–18.0)		78	59.0 (47.7–75.7)		25 (32.1%)	33 (42.3%)	20 (25.6%)	
Number of pregnancies														
1 time	1528	268 (17.5%)	858 (56.2%)	402 (26.3%)	<0.001	15.0 (12.5–20.0)	0.001	1528	70.9 (50.0–95.0)	0.673	377 (24.7%)	447 (29.3%)	704 (46.1%)	0.033
≥2 times	437	113 (25.9%)	239 (54.7%)	85 (19.5%)		15.0 (10.0–17.0)		437	71.0 (54.0–93.0)		89 (20.4%)	154 (35.2%)	194 (44.4%)	
Parity														
1 time	1584	282 (17.8%)	903 (57.0%)	399 (25.2%)	0.001	15.0 (12.0–20.0)	0.003	1584	71.7 (51.0–96.0)	0.656	369 (23.3%)	467 (29.5%)	748 (47.2%)	0.413
≥2 times	289	77 (26.6%)	154 (53.3%)	58 (20.1%)		15.0 (10.0–17.0)		289	72.0 (53.6–98.2)		61 (21.1%)	96 (33.2%)	132 (45.7%)	
Gestational age														
Premature infant	124	34 (27.4%)	56 (45.2%)	34 (27.4%)	0.017	15.0 (10.0–20.0)	0.124	124	72.2 (49.9–95.9)	0.865	31 (25.0%)	34 (27.4%)	59 (47.6%)	0.804
Full-term infant	1805	337 (18.7%)	1033 (57.2%)	435 (24.1%)		15.0 (12.0–19.0)		1805	71.1 (51.0–95.4)		427 (23.7%)	545 (30.2%)	833 (46.1%)	
Mode of delivery														
Spontaneous delivery	825	195 (23.6%)	460 (55.8%)	170 (20.6%)	<0.001	15.0 (11.0–17.5)	<0.001	825	71.4 (49.8–96.1)	0.800	207 (25.1%)	230 (27.9%)	388 (47.0%)	0.052
Caesarean section	1125	187 (16.6%)	626 (55.6%)	312 (27.7%)		15.0 (12.5–20.0)		1125	70.8 (52.8–93.5)		247 (22.0%)	368 (32.7%)	510 (45.3%)	
Birth weight														
<2500 g	98	21 (21.4%)	55 (56.1%)	22 (22.4%)	<0.001	15.0 (11.8–18.0)	<0.001	98	77.3 (54.0–97.0)	0.330	19 (19.4%)	28 (28.6%)	51 (52.0%)	0.146
2500–4000 g	1646	332 (20.2%)	934 (56.7%)	380 (23.1%)		15.0 (12.0–18.0)		1646	71.1 (54.0–95.3)		399 (24.2%)	490 (29.8%)	757 (46.0%)	
≥4000 g	221	26 (11.8%)	112 (50.7%)	83 (37.6%)		16.0 (13.8–20.0)		221	68.0 (53.5–90.6)		46 (20.8%)	82 (37.1%)	93 (42.1%)	
Regional distribution														
Nanjing	363	67 (18.5%)	177 (48.8%)	119 (32.8%)	<0.001	15.0 (12.0–20.0)	0.001	363	72.5 (61.0–93.8)	<0.001	45 (12.4%)	146 (40.2%)	172 (47.4%)	<0.001
Southern Jiangsu	478	76 (15.9%)	330 (69.0%)	72 (15.1%)		15.0 (12.5–20.0)		478	58.1 (27.9–87.0)		197 (41.2%)	104 (21.8%)	177 (37.0%)	
Central Jiangsu	376	81 (21.5%)	210 (55.9%)	85 (22.6%)		15.0 (12.5–17.0)		376	65.3 (44.3–92.0)		124 (33.0%)	106 (28.2%)	146 (38.8%)	
Northern Jiangsu	767	163 (21.3%)	389 (50.7%)	215 (28.0%)		15.0 (10.0–20.0)		767	77.2 (58.5–101.0)		104 (13.6%)	252 (32.9%)	411 (53.6%)	

Values are n (%) or median (P25–P75). GWG, gestational weight gain; reference category, 10 < GWG ≤ 19 kg. Vitamin D status: deficiency <50 nmol/L, insufficiency 50–<75 nmol/L, sufficiency ≥75 nmol/L. P values were derived from χ² and Kruskal–Wallis tests. Maternal demographic and perinatal characteristics were stratified by GWG categories and separately by offspring vitamin D status.

Conversely, factors associated with a significantly higher proportion of GWG ≤10 kg included: survey in winter, children aged 48–59 months, ≥2 pregnancies, preterm birth, spontaneous delivery, birth weight <2500 g, residence in Central Jiangsu, absence of calf cramps during pregnancy, initiation of vitamin D and calcium supplements within 5 gestational weeks, daily milk intake <200 mL, and fish/shrimp intake ≥4 times per week (*P* < 0.05). Household registration, offspring sex, and outdoor activity time during pregnancy exceeding one hour did not have a statistically significant impact on the distribution of GWG (*P* > 0.05).

### 3.2 The prevalence of vitamin D deficiency and vitamin D levels in offspring with different characteristics

A total of 1,984 offspring (52.2% boys, 47.8% girls) had a mean serum 25(OH)D level of 71.1 nmol/L. Levels declined with age and were significantly lower in spring and in the southern Jiangsu region. Lower offspring 25(OH)D levels were associated with maternal vitamin D and calcium supplementation during pregnancy, frequent fish and shrimp consumption ≥ 4 times per week, and limited outdoor exposure < 1 hour per day (**Table 3**).

**Table 3:** Maternal lifestyle during pregnancy stratified by gestational weight gain categories and separately by offspring vitamin D status.

Classification	N	Gestational weight gain						N	Offspring 25(OH)D, nmol/L		Offspring vitamin D levels and deficiency			
		GWG ≤10 kg	10 < GWG ≤ 19 kg	GWG >19 kg	*p*	*P50 (P25–P75)*	*p*		*P50 (P25–P75)*	*p*	VitD<50 nmol/L	50≤VitD<75 nmol/L	VitD≥75 nmol/L	*p*
		N (%)	N (%)	N (%)							N (%)	N (%)	N (%)	
Whether a mother’s calves cramp during pregnancy														
Yes	879	157(17.9%)	476(54.2%)	246(28.0%)	0.009	15.0(12.5-20.0)	0.002	879	72.0(51.7-96.4)	0.273	195(22.2%)	269(30.6%)	415(47.2%)	0.391
No	1086	226(20.8%)	619(57.0%)	241(22.2%)		15.0(12.0-18.0)		1086	70.0(50.0-92.6)		268(24.7%)	331(30.5%)	487(44.8%)	
Whether to take vitamin D and calcium supplements during pregnancy														
Yes	1192	250(21.0%)	629(52.8%)	313(26.3%)	0.003	15.0(12.0-20.0)	0.972	1192	69.5(49.9-94.3)	0.037	298(25.0%)	365(30.6%)	529(44.4%)	0.117
No	775	132(17.0%)	470(60.6%)	173(22.3%)		15.0(12.3-18.0)		775	73.5(53.0-94.4)		164(21.2%)	239(30.8%)	372(48.0%)	
Time for mothers to start taking vitamin D and calcium supplements during pregnancy														
Pre-pregnancy	14	2(14.3%)	9(64.3%)	3(21.4%)	0.010	15.0(11.8-18.5)	0.010	14	59.4(39.5-80.8)	0.063	6(42.9%)	4(28.6%)	4(28.6%)	0.036
0–5 weeks	131	34(26.0%)	56(42.7%)	41(31.3%)		15.0(10.0-20.0)		131	61.4(44.0-94.7)		46(35.1%)	33(25.2%)	52(39.7%)	
5–12 weeks	375	90(24.0%)	207(55.2%)	78(20.8%)		15.0(10.5-17.5)		375	70.4(50.0-94.4)		93(24.8%)	109(29.1%)	173(46.1%)	
>12 weeks	587	104(17.7%)	314(53.5%)	169(28.8%)		15.0(12.5-20.0)		587	72.2(52.7-96.0)		127(21.6%)	183(31.2%)	277(47.2%)	
Two classifications of mothers’ daily milk intake during pregnancy														
<200 mL	281	69(24.6%)	152(54.1%)	60(21.4%)	0.012	15.0(10.8-17.0)	0.001	281	68.4(51.5-86.2)	0.064	65(23.1%)	108(38.4%)	108(38.4%)	0.011
≥200 mL	1282	219(17.1%)	740(57.7%)	323(25.2%)		15.0(12.5-20.0)		1282	71.9(51.7-96.1)		299(23.3%)	382(29.8%)	601(46.9%)	
Frequency of weekly fish and shrimp intake during pregnancy in mothers														
<4 times/week	1314	244(18.6%)	724(55.1%)	346(26.3%)	0.045	15.0(12.5-20.0)	0.005	1314	73.2(54.0-96.4)	<0.001	273(20.8%)	407(31.0%)	634(48.2%)	<0.001
≥4 times/week	520	102(19.6%)	310(59.6%)	108(20.8%)		15.0(12.0-18.2)		520	65.0(47.0-89.3)		158(30.4%)	159(30.6%)	203(39.0%)	
Mothers’ daily outdoor activity time during pregnancy														
<1 h	330	59(17.9%)	201(60.9%)	70(21.2%)	0.111	15.0(12.0,18.0)	0.346	330	67.3(47.8-90.1)	0.014	94(28.5%)	96(29.1%)	140(42.4%)	0.044
≥1 h	1608	315(19.6%)	881(54.8%)	412(25.6%)		15.0(12.0,20.0)		1608	72.0(52.6-95.6)		356(22.1%)	498(31.0%)	754(46.9%)	

Values are n (%) or median (P25–P75). GWG, gestational weight gain; reference category, 10 < GWG ≤ 19 kg. Vitamin D status: deficiency <50 nmol/L, insufficiency 50–<75 nmol/L, sufficiency ≥uf nmol/L. P values were derived from χ² and Kruskal–Wallis tests. All maternal lifestyle variables refer to mothers during pregnancy. Maternal lifestyle characteristics were stratified by GWG categories and separately by offspring vitamin D status.

Among offspring, the proportions of vitamin D deficiency, insufficiency, and sufficiency were 23.7%, 30.6%, and 45.7%, respectively (**Table 4**). Vitamin D status differed significantly across GWG categories (*P* = 0.001). Specifically, the ≤10 kg GWG group exhibited the highest prevalence of vitamin D deficiency (30.5%), which was significantly higher than that in both the 10 < GWG ≤ 19 kg (22.1%) and >19 kg (22.0%) groups. Consistent with this pattern, the ≤10 kg group also had the lowest proportion of vitamin D sufficiency (43.2%), whilst the highest proportion (47.8%) was observed in the 10 < GWG ≤ 19 kg GWG group.

**Table 4:** Association between maternal gestational weight gain and offspring vitamin D status.

		Offspring vitamin D deficiency status					Offspring vitamin D concentration (nmol/L)	
Classification	N	VitD<50 nmol/L	50≤VitD<75 nmol/L	VitD≥75 nmol/L	χ^2^	*p*	*P50 (P25–P75)*	*p*
		N (%)	N (%)	N (%)				
Gestational Weight Gain								
GWG ≤10 kg	387	118 (30.5%)	102 (26.4%)	167 (43.2%)	17.761	0.001	68.2 (47.0, 93.2)	0.082
10 < GWG ≤ 19 kg	1106	244 (22.1%)	333 (30.1%)	529 (47.8%)			72.1 (51.8, 95.4)	
GWG >19 kg	491	108 (22.0%)	173 (35.2%)	210 (42.8%)			69.4 (53.3, 92.2)	

Values are n (%) or median (P25–P75). GWG, gestational weight gain; reference category, 10 < GWG ≤ 19 kg. Vitamin D status: deficiency <50 nmol/L, insufficiency 50–<75 nmol/L, sufficiency ≥75 nmol/L. P values were derived from χ² and Kruskal–Wallis tests.

### 3.3 Maternal GWG and offspring vitamin D deficiency/insufficiency: main and sensitivity analyses

As shown in **Figure 2**, exploratory curve fitting suggested a change in the association pattern at approximately 10 kg of GWG, with a significant non-linear association between GWG and offspring vitamin D levels (*P* = 0.028). We then examined the associations between GWG categories and offspring vitamin D outcomes using binary and multinomial logistic regression analyses (**Tables 1A**, **B**).

**Figure 2:**
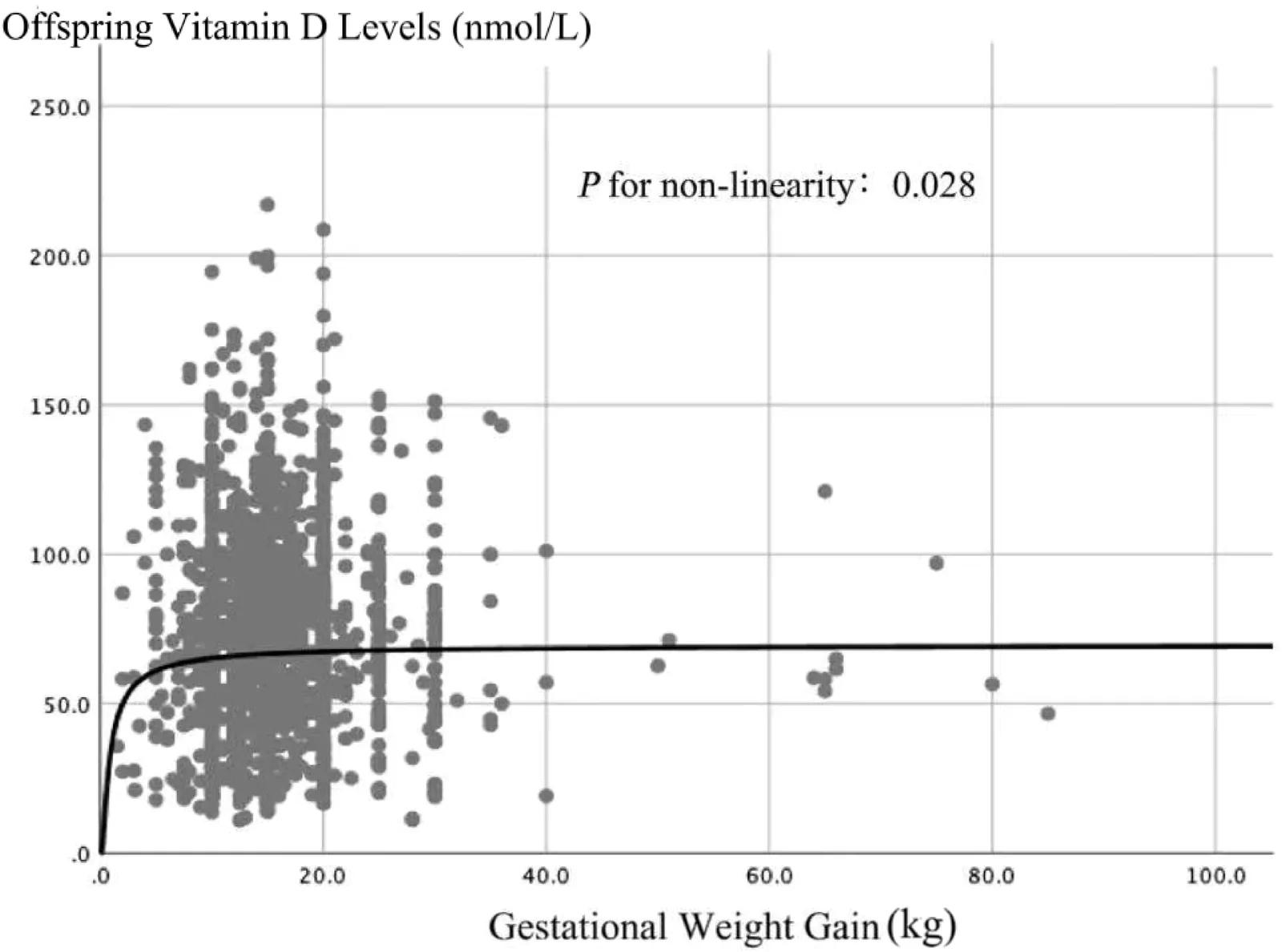
Exploratory curve fitting for the association between gestational weight gain and offspring vitamin D levels. The S-curve model provided the best fit (*P* for non-linearity, curve fitting = 0.028).

For vitamin D deficiency (<50 nmol/L vs. ≥50 nmol/L), GWG ≤10 kg was associated with a significantly higher risk compared with the reference group (10 < GWG ≤ 19 kg) in the unadjusted model (*OR* = 1.550, 95% CI: 1.196–2.008, *P* < 0.01). This association remained significant after adjustment for demographic characteristics (Model 1: *OR* = 1.820, 95% CI: 1.357–2.441, *P* < 0.01), maternal lifestyle factors during pregnancy (Model 2: *OR* = 1.545, 95% CI: 1.060–2.253, *P* < 0.05), and child lifestyle factors (Model 3: *OR* = 1.882, 95% CI: 1.228–2.883, *P* < 0.01) (**Table 1A**).

For vitamin D insufficiency (50–<75 nmol/L vs. ≥75 nmol/L), GWG >19 kg was associated with a significantly higher risk in the unadjusted model (*OR* = 1.309, 95% CI: 1.026–1.670, *P* < 0.05). This association remained significant after adjustment for demographic characteristics (Model 1: *OR* = 1.312, 95% CI: 1.021–1.685, *P* < 0.05), maternal lifestyle factors during pregnancy (Model 2: *OR* = 1.872, 95% CI: 1.305–2.684, *P* < 0.01), and child lifestyle factors (Model 3: *OR* = 1.609, 95% CI: 1.054–2.456, *P* < 0.05) (**Table 1B**). No significant association was observed between GWG ≤10 kg and vitamin D insufficiency, or between GWG >19 kg and vitamin D deficiency.

For the composite outcome of vitamin D deficiency or insufficiency (<75 nmol/L vs. ≥75 nmol/L), GWG >19 kg was not significantly associated with the outcome in the unadjusted model (*OR* = 1.208, 95% CI: 0.957–1.525, *P* > 0.05), but became significant after adjustment for demographic characteristics (Model 1: *OR* = 1.344, 95% CI: 1.067–1.693, *P* < 0.05) and maternal lifestyle factors during pregnancy (Model 2: *OR* = 1.472, 95% CI: 1.067–2.031, *P* < 0.05); the association was attenuated in Model 3 (*OR* = 1.278, 95% CI: 0.880–1.856, *P* > 0.05) (**Table 1A**).

In sensitivity analyses based on Model 3, FDR correction was applied using the Benjamini–Hochberg method. The association between GWG ≤WG kg and vitamin D deficiency remained significant after FDR correction (*q* = 0.027), whereas the association between GWG >19 kg and vitamin D insufficiency did not remain statistically significant after FDR correction (*q* = 0.056) (**Supplementary Tables S1a, b**).

To further validate the functional form without imposing a predefined shape, we performed RCS regression as a sensitivity analysis, with vitamin D deficiency as a binary outcome, adjusting for demographic characteristics (Model 1). As shown in **Supplementary Figure S1**, we did not observe a significant overall or nonlinear trend (*P* for overall = 0.255, *P* for nonlinearity = 0.140).

## 4 Discussion

In this multicentre retrospective cohort study of 1,984 mother–offspring pairs, to our knowledge, this is the first study to report an association between GWG ≤10 kg and an increased risk of vitamin D deficiency in offspring, and between GWG >19 kg and an increased risk of vitamin D insufficiency. However, the latter association did not remain significant after FDR correction in sensitivity analyses based on Model 3 and requires confirmation in further studies. These findings suggest that both insufficient and excessive GWG may be relevant to offspring vitamin D status, with the association for GWG ≤10 kg being more robust.

Previous studies have provided limited evidence on this topic. In a Montreal-based single-centre study, Mullahoo et al. classified mothers into inadequate, adequate, or excessive GWG categories according to Health Canada’s pre-pregnancy BMI-specific recommendations, and reported that neonates born to mothers with excessive GWG had lower 25(OH)D concentrations than those born to mothers with adequate GWG (ANOVA *P* = 0.02) (11). However, that study was a single-centre analysis with a small sample (n = 59) and compared GWG categories rather than identifying a specific quantitative threshold above which the risk increased. In contrast, our multicentre study used curve fitting to identify specific GWG thresholds (10 kg and 19 kg) and, to our knowledge, is the first multicentre study to quantitatively identify GWG thresholds associated with offspring vitamin D status, showing that GWG ≤10 kg was associated with vitamin D deficiency, whereas GWG >19 kg was associated with vitamin D insufficiency.

Several factors may explain why our findings extend those of Mullahoo et al. First, our larger sample size provided greater statistical power and allowed for adjustment for numerous confounders, whereas the Montreal study’s small sample (n = 59) may have been underpowered. Second, our study applied FDR correction and additional adjustment for child lifestyle factors, which may have attenuated the association. Third, GWG is a limited indicator of maternal obesity because it reflects increases in water and lean body mass, not just fat, which complicates the direct linkage between GWG and vitamin D storage in adipose tissue. Additionally, mothers with higher weight gain may adopt proactive measures, such as prenatal vitamin D supplementation, to mitigate the adverse effects of excessive GWG.

This study systematically analysed multiple factors influencing offspring vitamin D levels. Results indicate that maternal dietary and supplementation behaviours during pregnancy, including weekly consumption of fish and shrimp, daily outdoor activity duration, milk intake, and calcium and vitamin D supplementation, are all associated with offspring vitamin D levels. Building upon this foundation, the present study further adjusted for demographic characteristics and maternal lifestyle factors during pregnancy, aiming to more precisely evaluate the independent impact of maternal gestational weight gain on offspring vitamin D levels.

Because offspring vitamin D status is also influenced by child-level factors after birth, we further examined whether these factors altered the antenatal association. These factors have been described and analysed in our previous publication based on the same cohort. In our previous study (8), those not significantly associated with offspring vitamin D outcomes were not included, whereas the remaining child lifestyle factors were examined in Model 3. In Model 3, the association between GWG >19 kg and vitamin D insufficiency was statistically significant, but it did not remain significant after FDR correction. This association should therefore be considered exploratory and warrants further validation in future prospective studies.

RCS analysis did not reveal a significant nonlinear dose–response relationship between GWG and offspring vitamin D deficiency. This suggests that the observed association is better characterised by a threshold-like pattern, with the increased risk primarily concentrated amongst mothers with GWG ≤10 kg, rather than a progressive linear trend across the entire GWG range. The 10 kg threshold identified in the exploratory curve fitting should therefore be interpreted with caution and considered as exploratory rather than a definitive biological cut-off. Nevertheless, the between-group difference at the exploratory cut-point remained significant, and this finding should be interpreted as exploratory.

Numerous studies confirm that both low and excessive weight gain during pregnancy are clear risk factors for adverse birth outcomes. Inadequate weight gain raises the risk of small for gestational age (SGA) infants and preterm birth, whilst excessive weight gain increases the likelihood of large for gestational age (LGA) infants, macrosomia, and caesarean delivery. In Asian populations, certain risks associated with high weight gain may be even more pronounced (15–17). These clinical observations further support the “Developmental Origins of Health and Disease” hypothesis, suggesting that abnormal gestational weight gain may increase the offspring’s long-term risk of metabolic disorders through its programmatic effects on foetal development and metabolism (18, 19). Although vitamin D plays a crucial role in metabolic regulation, to our knowledge, only one single-centre study has examined the relationship between gestational weight gain and offspring vitamin D levels. The present study extends this evidence using a large multicentre cohort.

The potential mechanisms linking abnormal GWG to offspring health have been explored in previous studies. For insufficient GWG, maternal undernutrition impairs blood volume expansion and uteroplacental perfusion, compromising placental villous development, reducing nutrient exchange, and limiting foetal oxygen and nutrient supply (20). Maternal nutrient deficiency also downregulates placental amino acid transporters and disrupts insulin-like growth factor signalling, further restricting foetal nutrient availability and increasing SGA and low birth weight risk (21, 22). Additionally, maternal undernutrition induces gut microbiota dysbiosis in offspring, characterised by reduced microbial diversity and abundance, which may affect hepatic function and impair lipid metabolism (23, 24).

For excessive GWG, maternal adipose expansion drives a pro-inflammatory state with elevated leptin and decreased adiponectin. Hyperleptinemia programmes the developing hypothalamic appetite circuitry towards increased orexigenic drive, whilst hypoadiponectinemia induces insulin resistance via mTOR activation, collectively promoting fat accumulation and metabolic dysfunction (25, 26). Chronic low-grade inflammation, together with increased adipokine activity, further disrupts insulin signalling (27). Epigenetic reprogramming, including DNA methylation, histone modification, and altered non-coding RNAs, modifies expression of metabolic genes such as Ppargc1β and Fgf21, suppressing mitochondrial biogenesis, fatty acid oxidation, and brown adipose tissue development, culminating in reduced energy expenditure and enhanced white adiposity (28–31). Excessive GWG also alters placental structure and function, characterised by labyrinth layer thinning and abnormal fatty acid transport, promoting foetal adiposity (32, 33). Moreover, excessive GWG shifts maternal gut microbiota towards reduced abundance of short-chain fatty acid-producing bacteria, notably Ruminococcaceae, which may influence early gut colonization and subsequent metabolic programming in offspring (34, 35). However, these pathways were not directly examined in the present study, and our future research will further explore the mechanisms linking GWG to offspring vitamin D status.

Importantly, the association between GWG ≤10 kg and offspring vitamin D deficiency remained significant after sequential adjustment for demographic characteristics, maternal lifestyle factors during pregnancy, and child lifestyle factors. The persistence of this association suggests that GWG may play a role in offspring vitamin D status; however, given the observational nature of this study, this finding should be interpreted with caution and cannot be considered causal, and residual confounding from unmeasured factors such as pre-pregnancy BMI cannot be excluded. In contrast, the association between GWG >19 kg and vitamin D insufficiency was statistically significant in Model 3, but did not remain significant after FDR correction in sensitivity analyses, and residual confounding cannot be excluded.

This study possesses several key strengths, including its multicentre design, large sample size, and comprehensive multidimensional data collection. We systematically included various potential factors that could affect offspring vitamin D levels and thoroughly examined them in preliminary work, including demographic characteristics, maternal lifestyle factors during pregnancy, and child lifestyle factors. Leveraging this rich dataset, the study implemented robust adjustments for these factors. This enabled a more precise assessment of the association between gestational weight gain and offspring vitamin D levels, thereby enhancing the scientific rigour and reliability of the findings.

Nevertheless, several limitations should be acknowledged. First, data on maternal pre-pregnancy BMI were not available in our dataset. Pre-pregnancy BMI is a well-established determinant of both GWG and offspring metabolic outcomes, and its absence may introduce residual confounding. This limitation precludes us from fully disentangling the independent effect of GWG from that of maternal pre-pregnancy adiposity. Future prospective studies incorporating pre-pregnancy BMI are needed to validate our findings. Second, maternal serum vitamin D levels during pregnancy were not measured, which may have contributed to residual confounding. Third, as a retrospective study based on questionnaires, the results may be susceptible to recall bias. Fourth, although numerous confounders were accounted for, residual confounding due to unmeasured factors cannot be ruled out. Fifth, our data were collected in 2014–2015, and changes in population characteristics and clinical practices since then warrant caution when generalizing our findings to current populations.

These findings suggest that maintaining GWG within an appropriate range may be relevant to offspring vitamin D status; however, given the observational design, these findings should be interpreted as hypothesis-generating. Further prospective studies are needed to confirm these associations before any clinical recommendations can be made. Future research accounting for pre-pregnancy BMI and synchronising maternal and offspring vitamin D assessments is also warranted to better understand the underlying biological mechanisms.

## 5 Conclusion

To our knowledge, no multicentre study has previously reported an association between GWG ≤10 kg and an increased risk of vitamin D deficiency in offspring, and this association remained robust after adjustment for confounders. GWG >19 kg was associated with an increased risk of vitamin D insufficiency in the primary analysis; however, this association was no longer statistically significant after FDR correction in sensitivity analyses based on Model 3. It should therefore be interpreted as an exploratory finding warranting further validation in future prospective studies.

## Data Availability

The raw data supporting the conclusions of this article will be made available by the authors, without undue reservation.
