# Supplementary material for: Maternal gestational weight gain and offspring vitamin D level: a multicentre retrospective cohort study
Source: Front Endocrinol (Lausanne). 2026 Oct 8;17:1936050. doi: 10.3389/fendo.2026.1936050 (PMC13686902; doi:10.3389/fendo.2026.1936050)
Supplement: Supplementary file 1 [file DataSheet1.docx]

Supplementary Figure S1. Restricted cubic spline analysis for the association between gestational weight gain and offspring vitamin D deficiency. The outcome was defined as serum 25(OH)D < 50 nmol/L (vitamin D deficiency). The solid line represents the adjusted odds ratio, and the shaded area indicates the 95% confidence interval. The model was adjusted for demographic characteristics (Model 1), including household registration, child age group, number of pregnancies, investigation season, and regional distribution. The overall *P* value was 0.255, and the *P* for nonlinearity was 0.140. Note: In the main text (Table 2), vitamin D status was categorized into three groups (deficiency, insufficiency, and sufficiency). For this sensitivity analysis, the outcome was dichotomized as deficiency vs. non-deficiency to assess the threshold effect on the clinically relevant cut-off of <50 nmol/L.
