# Supplementary material for: Maternal gestational weight gain and offspring vitamin D level: a multicentre retrospective cohort study
Source: Front Endocrinol (Lausanne). 2026 Oct 8;17:1936050. doi: 10.3389/fendo.2026.1936050 (PMC13686902; doi:10.3389/fendo.2026.1936050)
Supplement: Supplementary file 2 [file Table1.docx]

**Supplementary Table**

| **Table S1a. Sensitivity analyses of the association between maternal GWG categories and offspring vitamin D outcomes using binary logistic regression** | | | | | | | |
| --- | --- | --- | --- | --- | --- | --- | --- |
| Outcome | sensitivity analysis | GWG ≤10 kg  OR (95%CI) | *P1* | *P1_FDR_q* | GWG >19 kg  OR (95%CI) | *P2* | *P2_FDR_q* |
| 25(OH)D < 50 nmol/L | Model 3 | 1.882 (1.228, 2.883) | 0.004 | 0.027 | 1.119 (0.706, 1.775) | 0.632 | 0.665 |
| 25(OH)D < 75 nmol/L | Model 3 | 1.278 (0.880, 1.856) | 0.198 | 0.264 | 1.349 (0.939, 1.937) | 0.105 | 0.205 |

Note: Reference category for the dependent variable: 25(OH)D ≥50 nmol/L for the <50 nmol/L outcome and 25(OH)D ≥75 nmol/L for the <75 nmol/L outcome. Reference group for maternal GWG: 10 < GWG ≤ 19 kg. *P1*, GWG ≤10 kg vs. reference; *P2*, GWG >19 kg vs. reference. *P1_FDR_q* and *P2_FDR_q* are FDR-corrected q-values (Benjamini–Hochberg method). Model 3 adjusted for child lifestyle factors, including daily milk intake (binary classification), timing of the first vitamin D supplementation after birth, age at first complementary feeding (months), vitamin D and calcium supplementation within the past 3 months, frequency of fish and shrimp intake, and child daily outdoor activity time.

**Table S1b. Sensitivity analyses of the association between maternal GWG categories and offspring vitamin D outcomes using multinomial logistic regression**

| Outcome | sensitivity analysis | GWG ≤10 kg  OR (95%CI) | *P1* | *P1_FDR_q* | GWG >19 kg  OR (95%CI) | *P2* | *P2_FDR_q* |
| --- | --- | --- | --- | --- | --- | --- | --- |
| 25(OH)D < 50 nmol/L | Model 3 | 1.948 (1.161, 3.268) | 0.012 | 0.048 | 1.346 (0.779, 2.325) | 0.286 | 0.381 |
| 50 ≤ 25(OH)D < 75 nmol/L | Model 3 | 1.127 (0.707, 1.796) | 0.616 | 0.616 | 1.609 (1.054, 2.456) | 0.028 | 0.056 |

Note: Reference category for the dependent variable: 25(OH)D ≥75 nmol/L (sufficiency). Reference group for maternal GWG: 10 < GWG ≤ 19 kg. *P1*, GWG ≤10 kg vs. reference; *P2*, GWG >19 kg vs. reference. *P1_FDR_q* and *P2_FDR_q* are FDR-corrected q-values (Benjamini–Hochberg method). Model 3 adjusted for child lifestyle factors, including daily milk intake (binary classification), timing of the first vitamin D supplementation after birth, age at first complementary feeding (months), vitamin D and calcium supplementation within the past 3 months, frequency of fish and shrimp intake, and child daily outdoor activity time.
